# Supplementary material for: Blue Light and Temperature Actigraphy Measures Predicting Metabolic Health Are Linked to Melatonin Receptor Polymorphism
Source: Biology (Basel). 2023 Dec 30;13(1):22. doi: 10.3390/biology13010022 (PMC10813279; doi:10.3390/biology13010022)
Supplement: Supplementary file 1 [file biology-13-00022-s001.zip › biology-2747582-supplementary.pdf]

Table S1. Reference values for blue light exposure (BLE) per time epoch, used to calculate Nocturnal Excess Index (grey shaded) and Daylight Deficiency Index (yellow shaded). Note that device measures BLE in Micro-watt/cm². https://www.unitsconverters.com was used to relate Micro-watt/cm² to Lux. Daylight maximum was set at 500 lux (taken that 250 lux at the eye * 2, considering approximate 2-fold difference between retina and desk table as actimeter is place on hand). References are from back-log transformation of raw references. Gray area references, though not yellow area references, are from cosine approximation of log_{10}.

Time Epoch Start	Microwatt/cm²	Lux	Raw Reference, Lux
00:00	0.032	0.217	0.250
00:30	0.029	0.201	0.000
01:00	0.029	0.200	0.000
01:30	0.031	0.212	0.000
02:00	0.035	0.241	0.000
02:30	0.043	0.293	0.000
03:00	0.055	0.377	0.000
03:30	0.075	0.514	0.000
04:00	0.108	0.738	0.000
04:30	0.162	1.110	0.000
05:00	0.254	1.734	3.000
05:30	0.409	2.793	10.000
06:00	0.674	4.603	30.000
06:30	1.126	7.693	55.000
07:00	1.893	12.929	100.000
07:30	3.170	21.650	145.000
08:00	5.243	35.808	190.000
08:30	8.492	57.998	235.000
09:00	13.358	91.235	280.000
09:30	20.251	138.312	324.000
10:00	29.376	200.641	368.000
10:30	40.518	276.739	412.000
11:00	52.845	360.928	456.000
11:30	64.875	443.099	500.000
12:00	74.708	510.259	500.000
12:30	80.503	549.833	500.000
13:00	81.070	553.708	500.000
13:30	76.289	521.052	500.000
14:00	67.151	458.639	456.000
14:30	55.412	378.461	412.000
15:00	43.005	293.725	368.000
15:30	31.529	215.343	324.000
16:00	21.951	149.927	280.000
16:30	14.604	99.745	235.000
17:00	9.349	63.854	190.000
17:30	5.803	39.636	145.000
18:00	3.521	24.051	100.000
18:30	2.107	14.389	55.000
19:00	1.254	8.563	27.500
19:30	0.749	5.113	10.000
20:00	0.453	3.091	10.000
20:30	0.279	1.909	8.500
21:00	0.178	1.213	7.000
21:30	0.117	0.800	5.500

22:00	0.081	0.552	4.000
22:30	0.059	0.400	2.500
23:00	0.045	0.307	1.000
23:30	0.037	0.250	0.500

Comments for Supplemental Table 1.

20:00-5:00. The phase from the moment after which the melanopic effect should be minimized (less than the threshold of 10 lux — at least 3 hours before the expected bedtime [7], 21 hours is the average value for DLMO (Dim Light Melatonin Onset). In at least 1 hour, it is optimal to reduce the level of melanopic light exposure to the intensity of melanopic light exposure that does not interfere with the production of endogenous melatonin. During the entire sleep period, the exposure of melanopic light exposure should not exceed 1 lux at eye level [7], however, taking into account the pronounced individual sensitivity characteristics [83], zero reference values are recommended during 5 hours of basic sleep (00:00-5:00) sleep.

Upon awakening, blue light stimulates activity and should reasonably increase gradually from 6:00 to 9:00 (cortisol acrophase). From this time during the working day, until 17:00 hours, the recommended level of blue light should not be lower than 250 lux [7], but taking into account circadian physiology, its smooth increase to 500 lux during the daytime is reasonable for optimal alertness (12:00-14:00 hours, approximate melatonin bathyphase) with a further gradual decrease. After 17:00 hours, it is recommended to reduce the exposure of blue light below the optimal 250 lux until the above threshold levels are reached by the time melatonin synthesis begins.

For the prototype, we have adopted scientifically based standards for optimal Human Centric Lighting dynamics within 24 hours [7] - based on the actually obtained blue light, which most actively affects the melanopsin receptors of retinal ganglion cells. They conclude that "in the daytime, the recommended minimum level of melanopic light exposure to the eyes is 250 lux, measured in a vertical plane at a height of ≈ 1.2 m (i.e. vertical illumination at eye level, in a sitting position). If possible, daylight should be used first to match these levels. If additional electric lighting is required, polychromatic white light should ideally have a spectrum, like natural daylight, enriched with shorter wavelengths close to the peak of melanopsin sensitivity.

Table S2. Comparative analysis of blue light exposure averaged over 30-min epochs in Arctic residents with BMI < 25 vs BMI > 25 (n = 62).

	BMI < 25 (n = 41)			BMI >25 (n = 21)			BMI < 25 +95% CI thresh-old	BMI > 25 −95%CI threshold
**Time epoch**	**Mean**	**−0.95**	**0.95**	**Mean**	**−0.95**	**0.95**		
08:45	8.500	5.167	13.981	18.830	12.589	28.166		
09:15	10.008	6.429	15.579	22.271	15.408	32.189		
09:45	11.080	6.432	19.085	25.398	17.972	35.893		
10:15	14.364	9.077	22.731	21.846	15.297	31.199		
10:45	14.837	9.784	22.499	28.179	18.680	42.509		
11:15	14.639	9.543	22.457	28.632	20.563	39.869		
11:45	14.223	8.452	23.933	29.005	19.609	42.904		
12:15	12.554	7.958	19.805	27.158	19.893	37.076		
12:45	14.555	9.742	21.746	32.912	22.925	47.249		
13:15	14.874	10.109	21.886	28.037	19.841	39.619		
13:45	11.304	7.316	17.466	31.385	20.913	47.102		
14:15	15.792	10.826	23.035	30.774	20.166	46.964		
14:45	15.232	10.676	21.731	23.116	17.095	31.256		
15:15	15.078	11.073	20.530	20.692	14.964	28.612		
15:45	14.028	9.965	19.747	17.342	12.275	24.501		
16:15	10.940	7.712	15.518	14.895	9.548	23.236		
16:45	8.584	5.763	12.787	13.502	8.751	20.833		
17:15	7.420	5.127	10.741	11.251	7.420	17.061		
17:45	6.322	4.526	8.832	6.756	4.240	10.764		
18:15	4.068	3.053	5.421	4.530	2.704	7.591		
18:45	2.631	1.976	3.502	3.507	2.254	5.457		
19:15	1.719	1.275	2.317	2.518	1.679	3.778		
19:45	1.265	0.928	1.725	2.131	1.315	3.454		
20:15	1.324	0.986	1.778	1.798	1.121	2.883		
20:45	1.008	0.726	1.401	1.765	1.268	2.456	**<1.5**	<1.5
**21:15**	**0.514**	**0.263**	1.003	**1.647**	1.160	**2.338**	**<1**	>1
**21:45**	**0.378**	**0.195**	0.732	**1.537**	1.137	**2.079**	**<0.75**	>1
**22:15**	**0.179**	**0.070**	0.460	**1.158**	0.752	**1.784**	**<0.5**	>0.75
**22:45**	**0.096**	**0.037**	0.248	**0.935**	0.515	**1.699**	**<0.25**	>0.5
**23:15**	**0.026**	**0.009**	0.077	**0.500**	0.228	**1.096**	**<0.1**	>0.2
**23:45**	**0.012**	**0.004**	0.034	**0.219**	0.090	**0.530**	**<0.05**	>0.9
**00:15**	**0.003**	**0.001**	0.009	**0.042**	0.011	**0.170**	**<0.01**	>0.01
00:45	0.002	0.001	0.005	0.012	0.003	0.055	0	0
01:15	0.001	0.000	0.002	0.003	0.001	0.015	0	0
01:45	0.001	0.000	0.001	0.002	0.000	0.006	0	0
02:15	0.000	0.000	0.001	0.001	0.000	0.005	0	0
02:45	0.000	0.000	0.001	0.001	0.000	0.002	0	0
03:15	0.000	0.000	0.001	0.000	0.000	0.002	0	0
03:45	0.001	0.000	0.002	0.001	0.000	0.003	0	0
04:15	0.000	0.000	0.001	0.001	0.000	0.004	0	0
04:45	0.001	0.000	0.003	0.004	0.001	0.017	0	0
05:15	0.008	0.003	0.021	0.013	0.003	0.051		
05:45	0.042	0.017	0.105	0.054	0.019	0.154		
06:15	0.167	0.066	0.424	0.314	0.157	0.631		

06:45	1.116	0.558	2.231	0.847	0.413	1.739
07:15	2.866	1.936	4.243	3.088	1.664	5.730
07:45	4.336	2.753	6.827	7.312	4.531	11.801
08:15	5.035	2.471	10.259	14.709	9.119	23.726

30-minute epochs of nocturnal excess when 95% confidence interval (CI) of the highest measured blue light exposure (μw/cm²) of BMI < 25 group does not overlap 95% CI of the lowest blue light exposure of BMI > 25 group are in **bold**. We show thresholds for epochs discriminating BMI groups in the right columns. **Suggested low threshold of nocturnal blue light exposure (20:30-05:00)** for optimal metabolic health is in **bold red** (BMI < 25 +95% CI threshold column).

Table S3. Actigraphy-derived characteristics depending on Body Mass Index (BMI) and MTNR1B G-allele in Arctic residents during spring equinox (n = 50).

Variable	MTNR1B genotype / BMI group			
	CC (n = 28)		CG + GG (n = 22)	
	BMI < 25 (n = 17)	BMI > 25 (n = 11)	BMI < 25 (n = 14)	BMI > 25 (n = 8)
**Activity, PIM**				
MESOR	2438 ± 597	2364 ± 534	2624 ± 468	2837 ± 1200
24-h A	1737 ± 385	1727 ± 473	1956 ± 474	1998 ± 823
Phase	14:27 ± 1:37	15:02 ± 1:02	15:17 ± 0:53	14:19 ± 1:17
M10	3877 ± 854	3876 ± 834	4221 ± 784	4594 ± 1955
M10 Onset	*8:31 ± 1:22*	8:59 ± 1:03	*9:47 ± 1:06*	8:35 ± 1:27
L5	245 ± 235	175 ± 106	272 ± 228	222 ± 290
L5 Onset	1:45 ± 1:14	1:39 ± 1:10	1:39 ± 1:12	1:10 ± 0:48
IV	0.890 ± 0.182	0.798 ± 0.126	0.865 ± 0.164	0.817 ± 0.162
IS	0.543 ± 0.104	0.535 ± 0.103	0.569 ± 0.118	0.508 ± 0.045
RA	0.870 ± 0.073	0.888 ± 0.051	0.888 ± 0.056	0.849 ± 0.093
CFI	0.660 ± 0.052	0.677 ± 0.043	0.682 ± 0.068	0.660 ± 0.044
**Wrist temperature**				
**MESOR**	**31.83 ± 0.36****	**31.74 ± 0.37***	**31.88 ± 0.46****	**31.10 ± 0.64**
24-h A	1.48 ± 0.55	1.28 ± 0.47	1.56 ± 0.49	1.30 ± 0.75
Phase	2:30 ± 1:32	2:21 ± 1:34	3:35:0:59	2:18 ± 2:16
**Sleep characteristics**				
Bedtime	22:42 ± 1:02	22:50 ± 1:08	22:41 ± 0:57	22:45 ± 1:30
Wake time	6:51 ± 1:08	7:14 ± 1:28	6:53 ± 0:52	6:59 ± 2:37
Sleep phase	2:47 ± 1:00	3:02 ± 1:07	2:47 ± 0:50	2:46 ± 1:44
Time in bed	8:07 ± 0:49	8:24 ± 1:23	8:12 ± 0:43	8:13 ± 2:29
Total sleep	7:13 ± 0:43	7:17 ± 1:23	7:15 ± 0:32	7:20 ± 2:25
Sleep latency, min	2.04 ± 1.20	3.10 ± 2.92	2.50 ± 2.02	3.23 ± 2.33
Sleep efficiency, %	88.64 ± 3.82	85.62 ± 7.42	87.68 ± 4.76	87.38 ± 5.95
WASO	0:50 ± 0:19	1:00 ± 0:31	0:53 ± 0:25	0:48 ± 0:26
**Blue light, μw/cm²**				
MESOR	11.07 ± 8.67	15.38 ± 9.40	9.90 ± 4.97	12.98 ± 4.33
24-h A	15.04 ± 11.05	23.14 ± 15.74	14.71 ± 7.41	18.41 ± 6.01
Phase	12:35 ± 0:44	13:01 ± 0:50	13:09 ± 0:40	12:45 ± 0:50
M10	20.67 ± 12.27	33.71 ± 22.78	21.60 ± 10.60	28.29 ± 10.24
M10 Onset	7:36 ± 0:46	8:02 ± 0:25	8:09 ± 0:46	7:38 ± 0:52
L5	0.10 ± 0.20	0.07 ± 0.16	0.06 ± 0.10	0.06 ± 0.10
L5 Onset	1:37 ± 2:21	1:12 ± 1:20	1:10 ± 1:21	1:45 ± 2:45
L5 $\log_{10}$	−1.99 ± 1.24	−2.13 ± 1.09	−2.35 ± 1.33	−2.09 ± 1.21
IV	0.909 ± 0.243	0.980 ± 0.333	0.960 ± 0.415	0.862 ± 0.341
IS	0.348 ± 0.116	0.416 ± 0.164	0.416 ± 0.143	0.373 ± 0.159
RA	0.979 ± 0.031	0.989 ± 0.015	0.992 ± 0.011	0.980 ± 0.021
DDI_{bl}	341 ± 119	275 ± 144	355 ± 90	300 ± 82
**NEI_{bl}**	2.34 ± 1.82	2.62 ± 1.45	**1.27 ± 1.11 ***	**4.38 ± 4.13**

All values in the groups are mean ± SD. 24 h A – 24-hour Amplitude; M10 -average value of 10 hours of greatest activity or blue light exposure; L5 – 5 hours of lowest activity or blue light exposure; IV – intra-daily variability; IS – inter-daily stability; RA – relative amplitude; DDI_{bl} – daylight deficiency index; NEI_{bl} – nocturnal excess index; CFI – circadian function index; Phases, Onset time are indicated in hh:mm; DDI_{bl} and NEI_{bl} in μw/cm²*hour; significant differences between the groups, Mann-Whitney U test are in **bold**. * $p < 0.05$; ** $p < 0.01$. vs. G-allele carriers of BMI > 25 group.

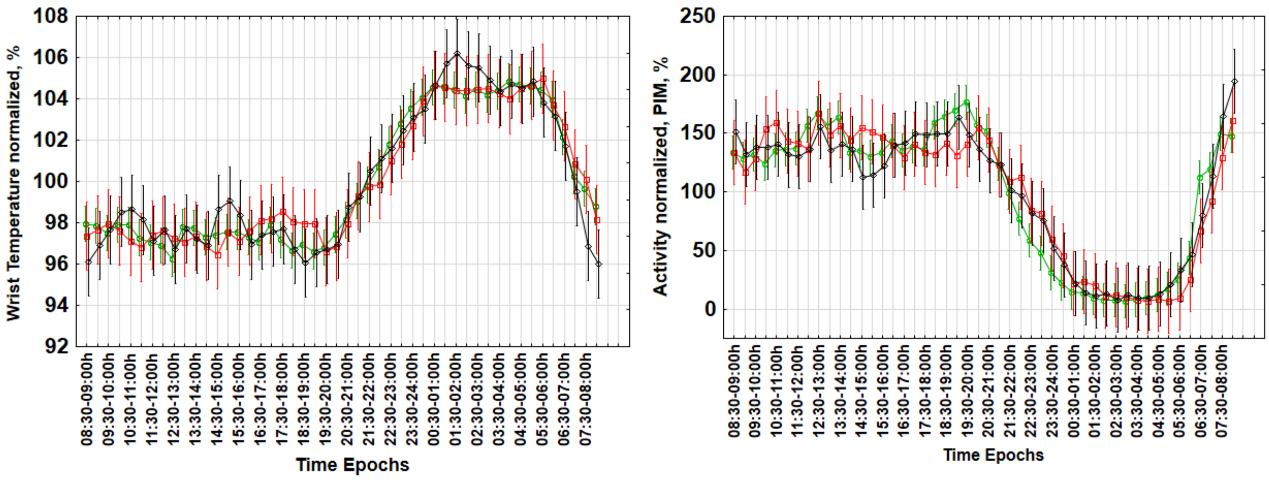

Figure S1. Similar amplitude and phase of wrist temperature and motor activity in Arctic residents with different body mass index (BMI). Vertical bars denote 95% confidence intervals. ANOVA for Time*group interaction; wrist temperature, $F_{(94, 2880)} = 0.727$, $p = 0.977$; motor activity = $F_{(94, 2880)} = 1.128$, $p = 0.192$. Normalized wrist temperature and activity in 30-minute time windows in groups with different BMI. green lines BMI <25; red lines BMI >25; black lines BMI >30.
